# Supplementary material for: GC-MS, GC-IMS, and E-Nose Analysis of Volatile Aroma Compounds in Wet-Marinated Fermented Golden Pomfret Prepared Using Different Cooking Methods
Source: Foods. 2024 Jan 25;13(3):390. doi: 10.3390/foods13030390 (PMC10855196; doi:10.3390/foods13030390)
Supplement: Supplementary file 1 [file foods-13-00390-s001.zip › foods-2806942-supplementary.pdf]

Table S1. Qualitative detection of volatile compounds in all samples by GC-IMS.

Compounds	CAS#	Formula	MW	RI	RT (s)	DT (ms)	Relative content					
							CK	A	B	C	D	E
Phenylacetaldehyde	C122781	C_8H_8O	120,2	1046,0	440,215	154,093	0.16±0.01^{a}	0.03±0^{c}	0.04±0.01^{b}	0.04±0.01^{b}	0.04±0.01^{b}	0.04±0.01^{b}
3-methylthiopropanal	C3268493	C_4H_8OS	104,2	908,9	283,801	109,067	0.11±0.04^{c}	0.07±0.01^{d}	0.14±0.02^{b}	0.10±0.01^{c}	0.24±0.03^{a}	0.10±0.01^{c}
Heptanal	C111717	$C_7H_{14}O$	114,2	902,1	278,14	169,604	0.03±0.01^{f}	0.08±0.02^{d}	0.14±0.04^{b}	0.04±0.01^{e}	0.22±0.01^{a}	0.11±0.01^{c}
2-Methyl-2-pentenal	C623369	$C_6H_{10}O$	98,1	836,7	235,456	149,453	0.29±0.10^{a}	0.06±0.02^{c}	0.09±0.01^{b}	0.08±0.01^{bc}	0.04±0.01^{d}	0.04±0.01^{d}
(E)-2-pentenal	C1576870	C_5H_8O	84,1	751,7	190,716	111,657	0.04±0.01^{e}	0.08±0.03^{b}	0.11±0.01^{a}	0.03±0.01^{f}	0.06±0.01^{c}	0.05±0^{d}
3-methylbutanal	C590863	$C_5H_{10}O$	86,1	656,8	153,612	140,305	12.08±0.23^{b}	13.72±0.73^{a}	2.58±0.45^{e}	9.54±0.63^{d}	11.79±0.92^{c}	11.91±0.82^{c}
Butanal	C123728	C_4H_8O	72,1	607,0	139,918	109,892	0.24±0.07^{e}	0.64±0.06^{b}	0.41±0.02^{c}	1.09±0.42^{a}	0.25±0.01^{e}	0.37±0.01^{d}
2-Methyl-2-propenal	C78853	C_4H_6O	70,1	574,0	131,526	105,954	2.69±0.14^{d}	3.44±0.28^{bc}	4.16±0.26^{a}	0.15±0.01^{e}	3.16±0.1^{c}	3.35±0.10^{b}
2-methylpropanal	C78842	C_4H_8O	72,1	542,7	124,017	129,308	0.33±0.16^{d}	2.20±0.39^{a}	0.24±0.03^{c}	0.15±0.01^{e}	0.96±0.28^{b}	0.97±0.22^{b}
Acroleine	C107028	C_3H_4O	56,1	482,4	110,765	118,038	0.44±0.07^{a}	0.29±0.01^{d}	0.33±0.03^{c}	0.19±0.04^{e}	0.36±0.06^{bc}	0.39±0.01^{b}
Methyl-5-hepten-2-one	C110930	$C_8H_{14}O$	126,2	987,6	357,857	116,371	0.24±0.03^{d}	0.38±0.03^{b}	0.49±0.05^{a}	0.18±0.03^{f}	0.37±0.01^{c}	0.29±0.02^{e}
2-heptanone	C110430	$C_7H_{14}O$	114,2	892,7	270,552	162,852	0.09±0.01^{c}	0.10±0.01^{b}	0.12±0.01^{ab}	0.05±0.01^{d}	0.13±0.01^{a}	0.12±0.01^{ab}
Mesityl oxide	C141797	$C_6H_{10}O$	98,1	796,2	213,008	143,755	0.27±0.11^{b}	0.11±0.03^{e}	0.21±0.01^{c}	0.85±0.22^{a}	0.15±0.01^{d}	0.11±0.01^{e}
2-Hexanone	C591786	$C_6H_{12}O$	100,2	781,0	205,103	119,383	0.20±0.01^{cd}	0.17±0.01^{d}	0.26±0.01^{b}	0.20±0.01^{cd}	0.33±0.02^{a}	0.21±0.02^{c}
Hydroxyacetone	C116096	$C_3H_6O_2$	74,1	712,6	173,047	122,926	1.46±0.13^{b}	0.57±0.21^{f}	0.78±0.01^{e}	1.94±0.11^{a}	1.21±0.12^{d}	1.26±0.02^{c}
3-hydroxybutan-2-one	C513860	$C_4H_8O_2$	88,1	715,1	174,151	133,245	9.13±0.41^{b}	5.04±1.23^{e}	5.2±0.61^{d}	12.55±1.39^{a}	3.52±0.32^{f}	5.53±0.17^{c}
2-Pentanone	C107879	$C_5H_{10}O$	86,1	686,7	162,446	137,182	2.63±1.01^{a}	0.57±0.11^{f}	1.4±0.08^{b}	0.62±0.13^{e}	0.94±0.05^{c}	0.72±0.1^{d}
1-penten-3-one	C1629589	C_5H_8O	84,1	682,3	161,121	132,566	0.27±0.02^{d}	1.49±0.56^{a}	0.75±0.07^{b}	0.15±0.02^{e}	0.45±0.04^{c}	0.73±0.03^{bc}
3-Pentanone	C96220	$C_5H_{10}O$	86,1	688,1	162,888	1,122	1.53±0.20^{ab}	1.56±0.13^{a}	0.76±0.08^{e}	1.06±0.15^{d}	1.12±0.09^{c}	1.34±0.06^{b}
2-Butanone	C78933	C_4H_8O	72,1	581,1	133,293	124,555	8.98±0.51^{a}	6.67±0.80^{d}	7.87±0.46^{b}	5.06±2.23^{e}	5.25±0.69	7.06±1.01^{c}
Acetone	C67641	C_3H_6O	58,1	537,9	122,912	111,928	2.30±0.90^{f}	5.40±0.32^{a}	3.88±0.19^{b}	2.45±0.36^{e}	2.77±0.28^{d}	3.59±0.25^{c}
Methyl butanoate	C623427	$C_5H_{10}O_2$	102,1	990,7	361,192	143,989	0.08±0.01^{d}	0.62±0.08^{bc}	0.71±0.10^{a}	0.09±0.01^{d}	0.63±0.02^{b}	0.45±0.02^{c}

Acetic acid hexyl ester	C142927	$C_8H_{16}O_2$	144,2	1008,4	383,699	141,853	0.09 ± 0.02^{f}	0.30 ± 0.07^{d}	0.43 ± 0.04^{b}	0.11 ± 0.01^{e}	0.50 ± 0.03^{a}	0.33 ± 0.01^{c}
Isobutyl butyrate	C539902	$C_8H_{16}O_2$	144,2	951,4	321,595	135,162	0.23 ± 0.08^{b}	0.05 ± 0.01^{e}	0.08 ± 0.01^{c}	0.28 ± 0.17^{a}	0.07 ± 0.01^{d}	0.08 ± 0.01^{c}
Propyl butanoate	C105668	$C_7H_{14}O_2$	130,2	893,9	271,5	126,558	0.70 ± 0.31^{c}	0.25 ± 0.03^{d}	0.22 ± 0.01^{e}	0.17 ± 0.01^{f}	1.04 ± 0.09^{a}	0.84 ± 0.01^{b}
1-methoxy-2-propyl acetate	C108656	$C_6H_{12}O_3$	132,2	859,4	249,052	115,479	0.34 ± 0.04^{b}	0.10 ± 0.03^{e}	0.10 ± 0.01^{e}	0.39 ± 0.08^{a}	0.28 ± 0.04^{c}	0.20 ± 0.01^{d}
Butyl formate	C592847	$C_5H_{10}O_2$	102,1	738,4	184,532	120,075	0.12 ± 0.02^{e}	0.16 ± 0.01^{c}	0.29 ± 0.01^{b}	0.13 ± 0.02^{d}	0.50 ± 0.03^{a}	0.29 ± 0.01^{b}
n-Propyl acetate	C109604	$C_5H_{10}O_2$	102,1	718,7	175,697	11,668	7.30 ± 1.85^{c}	4.60 ± 1.08^{f}	6.01 ± 0.53^{e}	6.43 ± 1.53^{d}	10.04 ± 0.83^{a}	8.33 ± 0.11^{b}
Ethyl acrylate	C140885	$C_5H_8O_2$	100,1	693,1	164,875	143,292	0.06 ± 0.02^{e}	0.21 ± 0.03^{d}	1.38 ± 0.28^{a}	0.06 ± 0.02^{e}	1.28 ± 0.05^{b}	0.31 ± 0.03^{c}
Acetic acid ethyl ester	C141786	$C_4H_8O_2$	88,1	607,0	139,918	133,924	0.34 ± 0.11^{c}	0.38 ± 0.15^{b}	0.15 ± 0.02^{d}	1.07 ± 0.54^{a}	0.08 ± 0.01^{f}	0.10 ± 0.01^{e}
Ethyl formate	C109944	$C_3H_6O_2$	74,1	622,0	143,894	106,633	0.69 ± 0.10^{f}	1.29 ± 0.12^{b}	1.11 ± 0.19^{c}	1.62 ± 0.26^{a}	0.88 ± 0.03^{e}	1.06 ± 0.01^{d}
Propyl propanoate	C106365	$C_6H_{12}O_2$	116,2	798,0	213,956	122,126	1.36 ± 0.41^{a}	0.30 ± 0.02^{f}	0.39 ± 0.03^{e}	0.62 ± 0.18^{b}	0.50 ± 0.05^{c}	0.44 ± 0.02^{d}
Pentanoic acid	C109524	$C_5H_{10}O_2$	102,1	909,0	283,831	12,181	0.05 ± 0.01^{bc}	0.03 ± 0.01^{d}	0.06 ± 0.01^{b}	0.04 ± 0.01^{c}	0.18 ± 0.01^{a}	0.06 ± 0.01^{b}
3-methylbutanoic acid	C503742	$C_5H_{10}O_2$	102,1	848,5	242,412	121,493	0.72 ± 0.15^{a}	0.17 ± 0.13^{c}	0.14 ± 0.01^{e}	0.38 ± 0.05^{b}	0.15 ± 0.04^{d}	0.14 ± 0.01^{e}
Butanoic acid	C107926	$C_4H_8O_2$	88,1	808,6	219,647	116,218	0.94 ± 0.08^{c}	0.40 ± 0.11^{f}	0.46 ± 0.02^{e}	1.36 ± 0.24^{a}	1.27 ± 0.22^{b}	0.77 ± 0.12^{d}
Propanoic acid	C79094	$C_3H_6O_2$	74,1	690,9	163,992	127,271	0.24 ± 0.11^{d}	0.14 ± 0.01^{e}	0.48 ± 0.02^{b}	0.14 ± 0.08^{e}	0.89 ± 0.11^{a}	0.31 ± 0.01^{c}
Acetic acid	C64197	$C_2H_4O_2$	60,1	626,9	145,219	114,101	0.88 ± 0.28^{b}	0.42 ± 0.10^{e}	0.61 ± 0.12^{d}	0.68 ± 0.27^{c}	1.04 ± 0.09^{a}	0.87 ± 0.02^{bc}
Formic acid	C64186	CH_2O_2	46,0	522,3	119,379	104,732	1.18 ± 0.52^{e}	1.65 ± 0.13^{c}	3.54 ± 0.29^{b}	3.78 ± 1.31^{a}	0.93 ± 0.14^{f}	1.43 ± 0.04^{d}
2-furanmethanethiol	C98022	C_5H_6OS	114,2	903,6	279,405	134,154	0.13 ± 0.03^{d}	0.55 ± 0.12^{c}	0.82 ± 0.12^{b}	0.11 ± 0.03^{e}	1.21 ± 0.04^{a}	0.81 ± 0.01^{bc}
Heptan-2-ol	C543497	$C_7H_{16}O$	116,2	892,7	270,552	136,053	0.39 ± 0.17^{c}	0.10 ± 0.01^{e}	0.12 ± 0.01^{d}	0.08 ± 0.02^{f}	0.75 ± 0.04^{a}	0.50 ± 0.02^{b}
n-Hexanol	C111273	$C_6H_{14}O$	102,2	873,5	257,905	132,782	0.15 ± 0.02^{e}	0.94 ± 0.21^{a}	0.25 ± 0.02^{c}	0.19 ± 0.03^{d}	0.18 ± 0.01^{de}	0.29 ± 0.01^{b}
Cyclohexanol	C108930	$C_6H_{12}O$	100,2	873,0	257,588	164,857	0.03 ± 0.01^{d}	0.23 ± 0.12^{a}	0.05 ± 0.01^{b}	0.04 ± 0.01^{c}	0.04 ± 0.01^{c}	0.04 ± 0.01^{c}
(E)-3-hexen-1-ol	C928972	$C_6H_{12}O$	100,2	837,2	235,772	12,297	1.09 ± 0.17^{a}	0.36 ± 0.28^{c}	0.16 ± 0.01^{e}	0.60 ± 0.06^{b}	0.18 ± 0.04^{d}	0.18 ± 0.03^{d}
2-Hexanol	C626937	$C_6H_{14}O$	102,2	794,4	212,059	128,246	0.46 ± 0.07^{f}	2.36 ± 0.11^{c}	2.94 ± 0.10^{a}	0.61 ± 0.12^{e}	2.55 ± 0.09^{b}	2.28 ± 0.04^{d}
Methyl-3-but-3-en-1-ol	C763326	$C_5H_{10}O$	86,1	733,6	182,323	140,713	4.95 ± 0.42^{b}	2.47 ± 0.14^{c}	1.53 ± 0.07^{d}	5.53 ± 0.23^{a}	0.62 ± 0.05^{f}	1.38 ± 0.07^{e}
1-Pentanol	C71410	$C_5H_{12}O$	88,1	763,2	196,237	151,982	0.06 ± 0.01^{d}	0.36 ± 0.11^{bc}	0.40 ± 0.08^{a}	0.04 ± 0.01^{e}	0.37 ± 0.03^{b}	0.34 ± 0.01^{c}
3-methylbutanol	C123513	$C_5H_{12}O$	88,1	734,1	182,544	150,624	3.82 ± 0.36^{a}	1.90 ± 0.71^{c}	1.01 ± 0.17^{d}	2.17 ± 0.75^{b}	0.28 ± 0.01^{f}	0.70 ± 0.04^{e}

1,2-Propanediol	C57556	$C_3H_8O_2$	76,1	735,5	183,207	128,357	0.58±0.13^{b}	0.48±0.03^{cd}	0.54±0.04^{c}	0.64±0.06^{a}	0.42±0.01^{e}	0.46±0.03^{d}
Ethylsulfide	C352932	$C_4H_{10}S$	90,2	710,0	171,943	107,448	1.82±0.11^{d}	2.88±0.29^{bc}	3.08±0.11^{a}	2.25±0.05^{c}	2.99±0.12^{b}	3.08±0.08^{a}
3-Methyl-2-butanol	C598754	$C_5H_{12}O$	88,1	688,8	163,109	122,655	1.03±0.30^{d}	0.64±0.11^{f}	1.74±0.13^{b}	0.75±0.22^{e}	2.22±0.19^{a}	1.29±0.04^{c}
1-butanol	C71363	$C_4H_{10}O$	74,1	668,2	156,925	117,631	0.87±0.05^{e}	1.21±0.06^{c}	1.02±0.06^{d}	1.62±0.18^{a}	1.23±0.14^{bc}	1.25±0.03^{b}
2-Butanol	C78922	$C_4H_{10}O$	74,1	635,7	147,648	132,295	1.08±0.10^{b}	1.16±0.05^{a}	0.60±0.06^{d}	1.05±0.06^{bc}	0.78±0.06^{cd}	0.92±0.05^{c}
2-methylpropanol	C78831	$C_4H_{10}O$	74,1	616,2	142,348	137,182	0.46±0.14^{a}	0.10±0.04^{bc}	0.06±0.01^{c}	0.11±0.01^{b}	0.05±0.01^{d}	0.05±0.01^{d}
1-Propanethiol	C107039	C_3H_8S	76,2	615,4	142,127	117,359	1.34±0.17^{a}	0.85±0.28^{b}	0.74±0.05^{c}	0.55±0.15^{f}	0.69±0.03^{d}	0.65±0.03^{e}
2-Propanethiol	C75332	C_3H_8S	76,2	576,7	132,188	115,866	2.19±0.70^{c}	1.40±0.38^{e}	1.99±0.36^{d}	1.90±0.74^{de}	4.66±0.54^{a}	3.49±0.01^{b}
β-Pinene	C127913	$C_{10}H_{16}$	136,2	964,7	334,516	129,041	0.06±0.01^{d}	0.08±0.02^{c}	0.18±0.02^{bc}	0.06±0.01^{d}	0.44±0.01^{a}	0.20±0.01^{b}
1-Pentene	C109671	C_5H_{10}	70,1	482,4	110,765	111,249	5.76±0.23^{c}	2.58±0.03^{e}	4.17±0.16^{d}	2.40±0.30^{f}	6.66±0.69^{b}	7.43±0.59^{a}
Styrene	C100425	C_8H_8	104,2	902,1	278,14	140,907	0.05±0.01^{d}	0.16±0.04^{c}	0.31±0.02^{bc}	0.06±0.01^{d}	0.63±0.04^{a}	0.34±0.01^{b}
Dipropyl disulfide	C629196	$C_6H_{14}S_2$	150,3	1105,7	547,321	148,793	0.34±0.08^{f}	0.56±0.05^{d}	0.69±0.04^{c}	0.47±0.17^{e}	0.97±0.07^{a}	0.74±0.04^{b}
Propylsulfide	C111477	$C_6H_{14}S$	118,2	898,6	275,294	116,112	0.20±0.03^{c}	0.15±0.02^{e}	0.20±0.03^{c}	0.17±0.02^{d}	0.23±0.02^{a}	0.21±0.01^{bc}
Diethylene glycol dimethyl ether	C111966	$C_6H_{14}O_3$	134,2	964,7	334,516	115,944	0.11±0.01^{d}	0.20±0.03^{c}	0.37±0.01^{b}	0.12±0.01^{d}	0.61±0.05^{a}	0.37±0.01^{b}
1-propene-3-methylthio	C10152768	C_4H_8S	88,2	695,8	165,98	104,461	0.32±0.08^{d}	0.39±0.04^{c}	0.41±0.06^{b}	0.94±0.19^{a}	0.27±0.01^{e}	0.38±0.03^{c}
4-methylthiazole	C693958	C_4H_5NS	99,2	792,6	211,111	134,787	0.28±0.01^{e}	1.50±0.23^{d}	2.57±0.15^{b}	0.47±0.07^{de}	2.78±0.21^{a}	1.97±0.07^{c}
Pyridine	C110861	C_5H_5N	79,1	758,6	194,029	125,098	1.46±0.04^{d}	2.14±0.27^{a}	1.55±0.08^{c}	1.65±0.08^{bc}	1.38±0.03^{e}	1.71±0.02^{b}
1,3-Dioxolane, 2,4-dimethyl, cis	C3390123	$C_5H_{10}O_2$	102,1	705,9	170,176	139,355	0.34±0.1^{c}	0.82±0.15^{a}	0.22±0.03^{d}	0.62±0.23^{b}	0.15±0.01^{f}	0.17±0.02^{e}
3-Butenenitrile	C109751	C_4H_5N	67,1	656,1	153,391	126,456	1.80±0.47^{d}	1.46±0.28^{e}	1.07±0.18^{f}	2.21±0.44^{c}	3.79±0.32^{a}	2.68±0.12^{b}
Dimethylamine	C124403	C_2H_7N	45,1	428,7	100,164	10,514	5.44±3.03^{e}	11.29±0.32^{c}	13.51±0.54^{b}	14.11±2.79^{a}	3.53±0.14^{f}	6.62±0.39^{d}
1					213,756		0.19±0.05^{a}	0.04±0.01^{b}	0.04±0.01^{b}	0.04±0.01^{b}	0.02±0.01^{d}	0.03±0.01^{c}
2					235,572		0.96±0.16^{a}	0.21±0.14^{d}	0.19±0.01^{e}	0.22±0.14^{cd}	0.38±0.06^{b}	0.23±0.04^{c}
3					194,329		0.68±0.18^{a}	0.21±0.06^{bc}	0.16±0.01^{d}	0.22±0.05^{b}	0.18±0.02^{c}	0.16±0.02^{d}

4	234,456	1.82±0.29^{b}	1.88±0.4^{a}	1.21±0.08^{c}	0.34±0.03^{f}	0.41±0.01^{e}	0.85±0.03^{d}
5	175,667	0.81±0.34^{d}	1.06±0.16^{b}	2.76±0.30^{a}	0.58±0.08^{f}	0.71±0.08^{e}	1.05±0.05^{c}
6	165,938	0.10±0.01^{f}	3.56±0.89^{c}	7.24±0.77^{a}	3.25±0.05^{d}	3.99±0.17^{b}	2.90±0.26^{e}

Note: 1 RT, retention time; DT, drift time; MW, molecular weight; RI, retention index. 2 different types of letters in the same row indicate significant difference ($P<0.05$)

Table S2. Detection of volatile compounds in all samples by GC-MS.

Compound	CAS	RI	CK	A	B	C	D	E
Hexanal	66-25-1	719	ND	13.44±3.04^{b}	12.37±2.11^{c}	ND	44.18±1.97^{a}	11.04±3.83^{d}
Isovaleric aldehyde	590-86-3	539	6.06±0.36^{d}	10.19±0.66^{c}	ND	19.64±4.89^{b}	20.87±0.13^{a}	ND
Nonanal	124-19-6	1094	1.99±0.6^{f}	9.12±1.02^{d}	12.1±7.14^{b}	4.43±0.98^{e}	13.96±4.91^{a}	11.52±6.02^{c}
Tetradecanal	124-25-4	1433	1.32±0.04^{c}	1.27±0.35^{d}	1.19±0.22^{f}	1.38±0.22^{b}	1.26±0.1^{e}	2.2±2.44^{a}
Pentadecanal	2765-11-9	1530	0.53±0.17^{f}	0.9±0.22^{d}	0.64±0.14^{e}	1.48±0.43^{b}	3.59±0.68^{a}	1.26±0.51^{c}
(2E)-Dodecenal	20407-84-5	1276	ND	ND	1.89±0.03^{a}	ND	0.07±0.01^{c}	0.15±0.01^{b}
Heptadecanal	629-90-3	1714	0.42±0.16^{c}	0.6±0.48^{b}	0.27±0.02^{e}	2.43±0^{a}	0.36±0.11^{d}	0.27±0.05^{f}
Benzaldehyde	100-52-7	1477	ND	2.02±0.35^{b}	0.92±0.51^{d}	0.53±0.09^{e}	3.66±0.29^{a}	1.36±0.56^{c}
Undecanal	112-44-7	1171	0.36±0.03^{d}	ND	1.45±0.23^{b}	0.93±0.04^{c}	ND	8.27±0.54^{a}
(2E)-Nonenal	18829-56-6	1157	ND	0.31±0.06^{d}	0.55±0.47^{b}	0.84±0.05^{a}	0.53±0.14^{c}	0.85±0.78^{a}
(2E)-2,4-Undecadienal	30361-29-6	1204	ND	ND	0.33±0.13^{a}	ND	ND	ND
(2E)-Octenal	2363-89-5	1112	0.68±0.09^{a}	ND	0.45±0.2^{b}	0.42±0.08^{b}	ND	ND
Icosanal	2400-66-0	2124	ND	ND	0.2±0.13^{a}	ND	ND	ND
(E)-2-Heptenal	18829-55-5	1291	ND	ND	ND	ND	0.67±0.13^{a}	ND
1-ethenyl-3-ethyl-Benzene	7525-62-4	1088	ND	0.66±0.48^{a}	ND	ND	0.32±0.02^{b}	ND
Octanal	124-13-0	1019	ND	ND	ND	ND	3.69±0.43^{a}	ND
(2E)-Octen-1-al	2548-87-0	1296	ND	0.45±0.01^{b}	ND	ND	0.85±0.02^{a}	0.39±0.12^{c}
(Z)-2-Decenal	2497-25-8	1168	ND	0.36±0.10^{b}	ND	0.37±0.21^{b}	0.56±0.12^{a}	ND
(2E)-2,4-Decadienal	25152-84-5	1002	ND	0.24±0.22^{c}	ND	ND	0.42±0.04^{b}	1.82±1.67^{a}
(E)-Tetradec-2-enal	51534-36-2	1427	ND	ND	ND	ND	0.31±0.03^{a}	ND
(9Z)-Hexadecenal	56219-4-6	1960	ND	ND	ND	0.43±0.13^{a}	0.37±0.21^{b}	ND
(Z)-7-Tetradecenal	65128-96-3	1445	ND	ND	ND	ND	0.36±0.04^{a}	ND
(E)-Decenal	3913-81-3	1167	0.18±0.01^{b}	ND	ND	ND	ND	1.42±1.81^{a}
Decanal	112-31-2	1129	ND	ND	ND	1.24±0.79^{b}	ND	1.61±0.10^{a}

3,5-Octadien-2-one	38284-27-4	1173	ND	ND	ND	ND	2.24±0.18^{a}	ND
2,3-Octanedione	585-25-1	1043	ND	ND	ND	ND	10.78±0.22^{a}	ND
5-methyl-5-Hepten-3-one	1190-34-7	929	ND	ND	0.67±0.05^{c}	2.6±0.34^{b}	6.24±0.67^{a}	ND
Acetoin	513-86-0	769	8.89±4.25^{b}	4.83±1.75^{d}	6.44±3.09^{c}	27.3±3.70^{a}	ND	2.69±0.91^{e}
Sageone	142546-15-4	1985	4.63±0.75^{a}	2.4±0.65^{b}	3.72±0.32^{ab}	2.02±0.02^{c}	ND	1.37±0.05^{bc}
6,10-dimethyl-5,9-Undecadien-2-one	689-67-8	1345	0.36±0.07^{b}	ND	ND	0.28±0.01^{c}	ND	2.61±0.2^{a}
2-Tridecanone	593-08-8	1656	ND	ND	ND	ND	ND	0.14±0^{a}
Tridecan-2-one	593-8-8	1197	2.15±0.07^{a}	ND	ND	ND	ND	ND
Heptan-2-one	110-43-0	965	3.33±0.48^{a}	ND	ND	1.62±0.02^{b}	ND	ND
2-Undecanone	112-12-9	1191	2.89±0.76^{a}	ND	ND	ND	ND	ND
2-Nonanone	821-55-6	1164	9.49±2.59^{a}	ND	ND	1.62±0.24^{b}	ND	ND
1-chloro-Octadecane	3386-33-2	1797	0.79±0.04a	ND	ND	ND	ND	ND
2,3-diethyl-Oxirane	4468-66-0	798	ND	ND	ND	ND	ND	5.69±0.89^{a}
Tridecane	629-50-5	1190	ND	ND	ND	ND	ND	1.88±1.22^{a}
Tetradecane	629-59-4	1403	1.6±0.12^{c}	ND	2.65±0.26^{a}	ND	ND	2.3±3.31^{b}
Pentadecane	629-62-9	1478	1.78±0.24^{e}	2.16±0.26^{d}	2.56±1.07^{a}	2.28±0.19^{c}	2.41±0.02^{b}	2.41±0.98^{b}
Hexadecane	544-76-3	1688	0.53±0.02^{e}	1.77±1.25^{a}	0.97±0.85^{b}	0.6±0.10^{d}	0.7±0.08^{c}	ND
Heptadecane	629-78-7	1789	ND	ND	1.17±0.73^{c}	ND	1.33±0.21^{b}	3.8±4.23^{a}
Cyclododecane	294-62-2	1221	ND	ND	0.66±0.36^{a}	ND	ND	ND
Octadecane	593-45-3	1688	ND	0.97±0.54^{a}	ND	ND	ND	ND
3-methyl-5-propyl-Nonane	31081-18-2	1302	ND	1.63±0.43^{a}	ND	ND	ND	ND
Farnesol	4602-84-0	1531	ND	ND	ND	ND	ND	0.27±0.04^{a}
2-ethyl-Hexanol	104-76-7	1166	ND	ND	ND	ND	ND	18.9±0.11^{a}

1-Octen-3-ol	3391-86-4	1136	ND	6.9±0.85[b]	5.55±1.56[c]	7.02±3.42[b]	7.17±0.19[a]	3.8±0.91[d]
Octanol	111-87-5	1221	1.48±0.12[a]	ND	0.57±0.1[c]	0.83±0.16[b]	ND	ND
Phenylethyl Alcohol	60-12-8	1478	10.9±1.67[a]	0.67±0.22[d]	1.62±0.37[c]	4.55±1.68[b]	0.34±0.03[e]	0.35±0.12[e]
Tridecanol	112-70-9	1434	ND	ND	ND	ND	0.55±0.1[b]	1.4±0.45[a]
(E)-2-Octen-1-ol,	18409-17-1	1461	5.01±0.11[a]	ND	ND	1.56±0.27[c]	2.02±0.84[b]	ND
(E)-2-Undecenol	37617-03-1	1230	2.17±0.32[a]	ND	ND	ND	ND	0.43±0.17[b]
2-methylpropionate4-Hexen-1-ol	42918-52-5	1214	0.3±0.18[a]	ND	ND	ND	ND	ND
1-Nonen-4-ol	35192-73-5	1148	ND	ND	ND	1.55±0.11[a]	ND	ND
β-Myrcene	123-35-3	1034	1.36±0.17b	ND	ND	3.42±0.05a	ND	ND
Limonene	138-86-3	1042	137.79±4.23[a]	ND	ND	ND	ND	ND
D-Limonene	5989-27-5	1042	ND	33.43±9.4[b]	28.75±3.36[c]	102.57±6[a]	23.76±2.08[d]	5.36±5.17[e]
1,3,5,7-Cyclooctatetraene	629-20-9	1083	ND	ND	0.72±0.25[b]	3.05±0.04[a]	ND	ND
Tridec-1-ene	2437-56-1	1303	ND	ND	0.29±0.02[b]	ND	ND	1.01±0.87[a]
Caryophyllene	87-44-5	1505	ND	0.6±0.06[b]	0.63±0.14[b]	ND	0.8±0.01[a]	ND
(Z)-Anethole	25679-28-1	1288	ND	ND	0.85±0.3[a]	ND	ND	ND
Styrene	100-42-5	1085	2.92±0.87[a]	0.61±0.05[c]	ND	2.92±0.07[a]	1.22±0.08[b]	ND
1-methyl-4-(1-methylethylidene)-Cyclohexene	586-62-9	1066	0.96±0.06[a]	0.48±0.08[c]	ND	ND	0.55±0.01[b]	ND
Tetradec-1-ene	1120-36-1	1487	0.36±0.01[b]	3.58±1.91[a]	ND	ND	0.38±0.18[b]	ND
Cumene	98-82-8	1103	ND	1.31±0.6[b]	ND	2.76±0.05[a]	ND	ND
Terpilene	99-86-5	1326	7.67±2.38[b]	3.36±0.31[c]	ND	10.21±1.96[a]	ND	ND
Azulene	275-51-4	1281	0.66±0.17[b]	0.71±0.03[a]	ND	0.68±0.09[ab]	ND	0.21±0.11[c]
Squalene	7683-64-9	2998	1.84±0.29[b]	2.31±0.58[a]	ND	1.51±0.39[c]	ND	ND
3,5,5-trimethyl-2-Hexene	26456-76-8	1138	2.09±0.41[a]	ND	ND	ND	ND	ND
Methyl 4-Methyl-2-oxopentanoate	3682-43-7	1129	2.85±0.39[a]	ND	ND	ND	ND	ND

Methyl 2-hydroxy-4-methylvalerate	40348-72-9	1179	7.47±0.71^{a}	ND	ND	ND	ND	ND
Glycidyl palmitate	7501-44-2	1918	ND	ND	ND	ND	0.29±0.05^{b}	0.32±0.11^{a}
2-Ketocaproic acid methyl ester	6395-83-1	1079	ND	ND	0.43±0^{a}	ND	ND	ND
Dimethyl-Phthalate	131-11-3	1256	ND	1±0.19^{d}	1.34±0.21^{b}	1.66±0.29^{a}	1.25±0.3^{c}	ND
Diisobutyl phthalate	84-69-5	1626	1.57±0.05^{b}	0.41±0.38^{e}	0.53±0.14^{d}	4.84±3.78^{a}	0.31±0.08^{f}	1.46±1.49^{c}
Dibutyl phthalate	84-74-2	1629	0.66±0.19^{c}	ND	0.62±0.16^{c}	0.78±0.53^{b}	0.25±0.05^{d}	1.08±0.3^{a}
2,2,4-Trimethyl-1,3-pentanediol diisobutyrate	6846-50-0	1609	ND	ND	ND	0.38±0.07b	ND	4.18±0.07a
3,6-Nonadien-1-yl acetate	76649-26-8	1302	0.38±0.14^{a}	ND	ND	ND	ND	ND
γ-Decalactone	706-14-9	1342	1.86±0.71^{a}	ND	ND	0.48±0.2^{b}	ND	ND
Acetic acid	64-19-7	974	ND	ND	0.69±0.01^{b}	ND	ND	1.48±0.08^{a}
Propionic acid	79-09-4	1075	ND	ND	0.71±0.07^{b}	ND	ND	1.09±0.41^{a}
Octanoic acid	124-7-2	1543	0.34±0.06^{b}	0.3±0.04^{b}	0.75±0.66^{a}	ND	ND	ND
Nonanoic acid	112-5-0	1338	0.21±0.04f	0.48±0.38d	0.54±0.25c	0.42±0.06e	1.03±0.13b	2.63±3.34a
Pentadecylic acid	1002-84-2	1654	ND	ND	1.86±1.53^{b}	4.57±1.42^{a}	1.28±0.01^{c}	0.34±0.21^{d}
Tetradecanoic acid	544-63-8	1457	2.06±0.18^{d}	2.72±0.18^{b}	2.28±1.26^{c}	1.6±0.02^{f}	1.97±0.36^{e}	3.39±1.95^{a}
Pentadecanolide	106-2-5	1544	ND	ND	ND	ND	ND	2.64±0.88^{a}
Stearic acid	1975-11-4	1876	ND	ND	ND	ND	ND	1.34±0.3^{a}
Toluene	108-88-3	789	2.9±1.31^{a}	ND	1.53±0.11^{b}	ND	ND	ND
N,N-dimethyl-Benzenamine	121-69-7	1080	ND	ND	0.5±0.08^{a}	ND	ND	ND
1,2,4,5-tetramethyl-Benzene	95-93-2	1107	0.81±0.42^{e}	1.04±0.09^{a}	0.88±0.41^{c}	0.98±0.49^{b}	0.97±0.03^{b}	ND
Estragole	140-67-0	1272	31.11±4.17^{a}	11.94±4.04^{d}	18.91±0.92^{b}	17.32±1.34^{c}	8.63±1.62^{e}	4.89±2^{e}
Phenol	108-95-2	1532	1.17±0.2^{c}	0.6±0.27^{f}	2.58±0.78^{a}	1.03±0.42^{d}	0.87±0.08^{e}	1.67±1.96^{b}
Butylated Hydroxytoluene	128-37-0	1446	79.54±5.62^{c}	78.43±6.42^{d}	95.07±23.65^{a}	81.06±8.87^{b}	71.67±3.15^{e}	62.91±2.82^{f}
Ethylbenzene	100-41-4	989	0.63±0.2^{a}	ND	ND	ND	0.44±0.05^{b}	ND

1,3-dimethyl-Benzene	108-38-3	934	ND	ND	ND	2.01±0[a]	0.67±0.03[b]	ND
1-ethyl-3-methyl-Benzene	620-14-4	1299	1.07±0.18[c]	ND	ND	2.52±0.67[a]	2.38±0.28[b]	ND
Mesitylene	108-67-8	1016	ND	ND	ND	1.24±0.09[b]	1.68±0.2[a]	ND
4-ethyl-1,2-dimethyl-Benzene	934-80-5	1320	0.39±0.07[e]	1.1±0.62[b]	ND	0.72±0.18[d]	0.78±0.05[c]	6.12±0[a]
3,5-bis(1,1-dimethylethyl)-Phenol	1138-52-9	1445	ND	ND	ND	ND	ND	0.56±0.5[a]
Benzopyridine	119-65-3	1091	0.42±0.07[ab]	ND	0.43±0.38[a]	ND	ND	0.41±0.09[b]
6-Caprolactam	105-60-2	1484	0.58±0.25[b]	ND	0.3±0.22[c]	0.91±0.18[a]	ND	ND
1-Dodecen-3-yne	74744-36-8	1260	ND	ND	1.03±0.04[b]	ND	ND	1.25±0.07[a]
Niacinamide	98-92-0	1876	ND	ND	0.19±0.01[b]	ND	ND	0.49±0.01[a]
Indole	120-72-9	1743	0.4±0.18[d]	0.76±0.19[b]	0.89±0.29[a]	0.68±0.14[c]	ND	ND
2-pentyl-Furan	3777-69-3	1015	ND	ND	ND	1.63±0.74[b]	2.05±0.17[a]	1.38±0.07[c]
1-(1H-pyrrol-2-yl)-Ethanone	1072-83-9	1405	ND	ND	ND	ND	0.54±0.23[a]	ND
1-Azanaphthalene	91-22-5	1294	ND	0.36±0.06[b]	ND	0.49±0.03[a]	ND	ND

Note:1 RI, retention index; 2 ND, no detected; different types of letters in the same row indicate significant difference (P<0.05)

Table S3. Odour thresholds and aroma-active compounds in different samples (supplementary material)

NO	Compound	Structure
A1	Hexanal	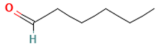
A2	Isovaleric aldehyde	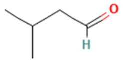
A3	Nonanal	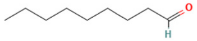
A4	(2E)-Dodecenal	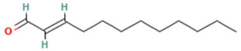
A5	Undecanal	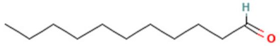
A6	(2E)-Nonenal	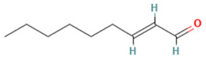
A7	(E)-2-Dodecenal	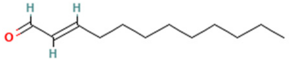

A8	Oct-2-enal	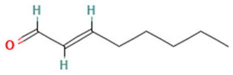
A9	(2E)-2-Octenal	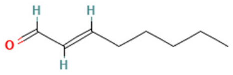
A10	(2E)-2,4-Decadienal	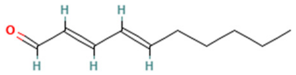
A11	(E)-Decenal	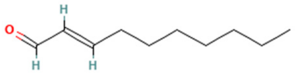
A12	Decanal	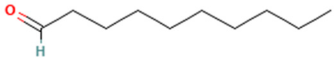
A13	Acetoin	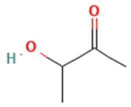
A14	Heptan-2-one	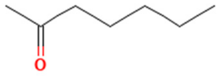
A15	2-Undecanone	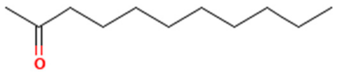

A16	2-Nonanone	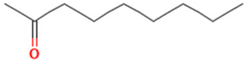
A17	1-Octen-3-ol	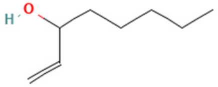
A18	(E)-2-Octen-1-ol	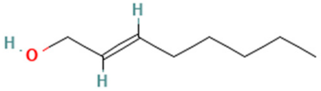
A19	β-Myrcene	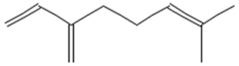
A20	Limonene	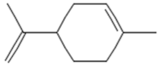
A21	D-Limonene	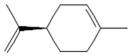
A22	Terpilene	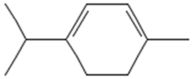
A23	γ-Decalactone	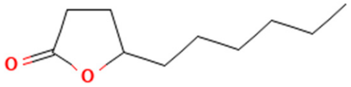

A24	Estragole	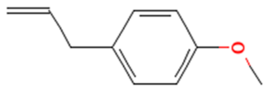
A25	2-pentyl-Furan	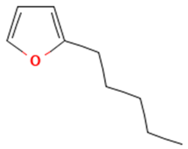
